# Plasma levels of osteopontin identify patients at risk for organ damage in systemic lupus erythematosus

**DOI:** 10.1186/ar4150

**Published:** 2013-01-23

**Authors:** Ornella J Rullo, Jennifer MP Woo, Miriam F Parsa, Alice DC Hoftman, Paul Maranian, David A Elashoff, Timothy B Niewold, Jennifer M Grossman, Bevra H Hahn, Maureen McMahon, Deborah K McCurdy, Betty P Tsao

**Affiliations:** 1 Department of Pediatrics-Rheumatology, David Geffen School of Medicine, University of California, Los Angeles, 10833 Le Conte Avenue, MDCC 12-430, Los Angeles, CA, 90095, USA; 2 Department of Medicine-Rheumatology, David Geffen School of Medicine, University of California, Los Angeles, 1000 Veteran Avenue, Rehabilitation Building 32-59, Los Angeles, CA, 90095, USA; 3 Department of Medicine-General Internal Medicine and Health Services Research, David Geffen School of Medicine, University of California, Los Angeles, 911 Broxton Avenue, Los Angeles, CA, 90095, USA; 4 Division of Rheumatology and Department of Immunology, Mayo Clinic, 200 1st Street SW, Rochester, MN 55905, USA

## Abstract

**Introduction:**

Osteopontin (OPN) has been implicated as a mediator of Th17 regulation via type I interferon (IFN) receptor signaling and in macrophage activity at sites of tissue repair. This study assessed whether increased circulating plasma OPN (cOPN) precedes development of organ damage in pediatric systemic lupus erythematosus (pSLE) and compared it to circulating plasma neutrophil gelatinase-associated lipocalin (cNGAL), a predictor of increased SLE disease activity.

**Methods:**

cOPN and cNGAL were measured in prospectively followed pSLE (*n *= 42) and adult SLE (aSLE; *n *= 23) patients and age-matched controls. Time-adjusted cumulative disease activity and disease damage were respectively assessed using adjusted-mean SLE disease activity index (SLEDAI) (AMS) and SLICC/ACR damage index (SDI).

**Results:**

Compared to controls, elevated cOPN and cNGAL were observed in pSLE and aSLE. cNGAL preceded worsening SLEDAI by 3-6 months (*P *= 0.04), but was not associated with increased 6-month AMS. High baseline cOPN, which was associated with high IFNalpha activity and expression of autoantibodies to nucleic acids, positively correlated with 6-month AMS (r = 0.51 and 0.52, *P *= 0.001 and 0.01 in pSLE and aSLE, respectively) and was associated with SDI increase at 12 months in pSLE (*P *= 0.001). Risk factors for change in SDI in pSLE were cOPN (OR 7.5, 95% CI [2.9-20], *P *= 0.03), but not cNGAL, cumulative prednisone, disease duration, immunosuppression use, gender or ancestry using univariate and multivariate logistic regression. The area under the curve (AUC) when generating the receiver-operating characteristic (ROC) of baseline cOPN sensitivity and specificity for the indication of SLE patients with an increase of SDI over a 12 month period is 0.543 (95% CI 0.347-0.738; positive predictive value 95% and negative predictive value 38%).

**Conclusion:**

High circulating OPN levels preceded increased cumulative disease activity and organ damage in SLE patients, especially in pSLE, and its value as a predictor of poor outcome should be further validated in large longitudinal cohorts.

## Introduction

The rapid and early accrual of organ damage in patients with systemic lupus erythematosus (SLE) has been associated with poor outcomes and increased mortality [1-3]. Patients with pediatric-onset SLE (pSLE; age of diagnosis < 18 years) are an important subset for the study of disease progression. In children and adolescents with SLE, organ damage is accumulated at increased rates compared with adults: childhood and adolescent-onset SLE has been associated with increased baseline renal and neuropsychiatric involvement, with more renal damage overall and higher mortality rates [4,5].

Despite many advances in the care of pSLE, our knowledge of potential noninvasive markers or predictors of irreversible injury is limited. Specifically studied in pSLE, clinical factors, such as adjusted mean SLE disease activity index (AMS) and corticosteroid use, are predictive of disease-related organ damage [6]. Additionally, neutrophil gelatinase-associated lipocalin (NGAL) has been reported not only as a useful biomarker for increased global and renal disease activity but also a predictor of impending renal flare in pSLE [7,8]. Other promising biomarkers for prediction of lupus nephritis flare in pSLE cohorts include transferrin, alpha1-acid-glycoprotein, and lipocalin-type prostaglandin-D synthetase, and in adult SLE (aSLE) cohorts, monocyte chemotactic protein-1, macrophage colony stimulating factor, regulated on activation, normal T expressed and secreted and hepcidin isoforms [9-13].

Osteopontin (OPN), a pluripotent secreted and intracellular phosphoprotein, has recently been implicated in the pathogenesis of autoimmunity. Although OPN has not been confirmed as an SLE-susceptibility gene, genetic variants in *OPN *have been associated with increased IFN-α pathway activation in young people with SLE, and with specific clinical phenotypes [14,15]. Intracellular OPN promotes expression of IFN-α in murine plasmacytoid dendritic cells, and contributes to Th17 cell commitment, which may be negatively regulated by IFN-α receptor signaling [16,17]. In support of the role of OPN in SLE pathogenesis, humans with SLE and autoimmune-prone mice (MRL-lpr/lpr) have increased OPN peripherally and in diseased tissue, which correlates with disease activity in humans [18,19].

When expressed by macrophages and activated T cells, OPN emerges as a key regulator of tissue repair in the context of inflammation [20-22]. Deficits in expected macrophage accumulation have been noted in OPN-null mice when challenged with chronic inflammatory conditions, such as granulomatous disease [23,24]. Growing evidence indicates that OPN splicing variants can provide diagnostic and/or therapeutic targets for several cancers due to functional diversity among OPN isoforms, particularly in terms of increased migratory and metastatic potential [25-29]. Importantly, in murine lupus nephritis, a specialized subset of macrophages known as alternatively activated macrophages, which participate in tissue repair, express OPN and mediate aggressive proliferative lesions with enhanced crescent formations [30]. OPN levels are associated with atherosclerosis and coronary artery disease, and in these plaques, serve as a chemotactic agent for infiltrating macrophages [31-34]. Phosphorylation of OPN is required for integrin binding and subsequent induction of IL-12 expression in macrophages, and for mediating their activation and spreading [23,35]. The phosphorylation status of OPN is at least in part mediated by tartrate-resistant acid phosphatase (TRAP), an enzyme inversely correlated with disease activity in pSLE [36]. Alternatively activated macrophages have similarly been associated with asthma-related airway remodeling, dermatomyositis skin lesions, and scleroderma-associated lung fibrosis, implicating a role in organ damage [37-39].

Based on the potential critical roles of OPN in IFN-α pathway signaling and dysregulated post-inflammatory tissue repair, we hypothesized that increased circulating plasma OPN (cOPN) levels precede the development of organ damage in pSLE and possibly aSLE. We compared cOPN with circulating plasma NGAL (cNGAL), a marker of pSLE disease flare, to investigate the potential for baseline cOPN to predict cumulative disease activity and organ damage. A biomarker of future disease course, particularly in pSLE, which is associated with more aggressive disease, could provide useful information for the clinical management of SLE.

## Materials and methods

### Subjects

All pSLE patients up to age 21 years who attended the clinic at the University of California, Los Angeles (UCLA) between September 2007 and January 2010 were offered the opportunity to enroll in the prospective cohort study. Children and young adults, fulfilling at least four of eleven of the American College of Rheumatology (ACR) 1997 revised classification criteria for SLE prior to age 18 years (defined as pSLE) were followed during routine visits at pediatric rheumatology clinics (*n *= 42) [40]. At each study visit, anti-dsDNA antibodies, complement (C)3 and C4 levels, erythrocyte sedimentation rate (ESR), serum creatinine, and urine analysis and microscopy were measured in the UCLA clinical laboratory, using standard methods. Antibodies to extractable nuclear antigens were measured at study entry. Healthy, unrelated age-matched (± 5 years) children and adolescents recruited from the UCLA pediatric clinics and the undergraduate campus served as controls (*n *= 22). All research procedures were conducted with the approval of the UCLA Institutional Review Board. All the patients who participated in the study, in addition to a parent or guardian in the case of individuals who were minors at the time of enrollment, signed appropriate consent documents detailing their voluntary participation in the study and permission to publish de-identified results from the research proceedings.

Samples obtained from an independent aSLE cardiovascular risk cohort with at least 12 months of complete prospective follow up were analyzed (if the samples contained an adequate amount of plasma), and clinical data and scored outcome measures were also studied [41]. All aSLE samples were from patients who consented to share samples for other SLE research and were seen by physicians in the UCLA Division of Rheumatology-Medicine (*n *= 23). All adult control samples (*n *= 40) were enrolled in the UCLA SLE genetics study, and were matched for age and gender with the adult SLE samples [42].

Patient medical records were reviewed for any kidney biopsy classification. At our center, kidney biopsies are performed on all patients with pSLE who have abnormal findings on urinalyses that cannot be explained by non-SLE-related mechanisms. The UCLA renal pathologists currently utilize the International Society of Nephrology/Renal Pathology Society (ISN/RPS) system of renal biopsy classification in lupus nephritis [43].

### Osteopontin and NGAL assays

Baseline peripheral blood samples from each participant were collected for intact plasma OPN and NGAL testing using ELISA (RnD Systems, Minneapolis, MN, USA). The inter- and intra-assay coefficients of variation were 5 to 10%. All measurements were made in duplicate and conducted by an investigator who was blinded to the identity of the subject. The plasma OPN includes both secreted OPN and any intracellular OPN that has been released by dying cells.

We define high cOPN as any value above the bottom three quartiles of healthy controls (*n *= 62; quartiles utilized as values are skewed to the right in the adult cohort) and also perform the high cOPN analyses utilizing a cutoff of two SD above the mean for young, healthy controls (mean age 20.2 years; *n *= 22). Our healthy, young unrelated controls most likely represent close-to-true normal values of cOPN, given the Gaussian distribution of the levels in that group.

### Reporter cell assay for interferon-alpha

A reporter cell assay for IFN-*α *activity was used as previously described [44,45]. Results from the IFN-*α *assay were standardized to a healthy multi-ancestral reference population as previously described, and a serum IFN-*α *activity score was calculated based upon the mean and SD of the reference population [44].

### Disease activity and damage

Global SLE disease activity was measured using the Safety of Estrogens in Lupus Erythematosus: National Assessment (SELENA) version of the SLE Disease Activity Index (SLEDAI; range 0 to 105). Renal disease activity was assessed using the renal component of the SLEDAI score; flare was assessed using the SELENA flare instrument. Cumulative disease activity (adjusted-mean SLEDAI, or AMS) was calculated by measuring the area under the curve of serial SLEDAI measurements [46]. Organ damage was measured by the Systemic Lupus International Collaborating Clinics/American College of Rheumatology SLE Damage Index (SDI; range 0 to 47). No SDI was calculated at study enrollment if: (a) diagnosis was less than 6 months prior to the time of blood draw; or (b) available medical records were inadequate to assess SDI at enrollment, due to transfer of care, et cetera (6 months of clinical data is required to accurately calculate SDI).

### Longitudinal study design

Patients with pSLE were followed prospectively every 3 months and at the time of disease flare for at least 12 months. Blood draws for cOPN and cNGAL measurement were obtained at baseline. SLEDAI and laboratory parameters were recorded at 0, 3, 6, 9 and 12 months, and at disease flare, and SDI was determined at 6 and 12 months. Only pSLE patients with one year of complete follow up at UCLA subsequent to study enrollment were included in the study. Patient samples and clinical data, including SLEDAI and SDI, from the prospectively collected UCLA aSLE cohort were used for confirmation and expansion of the results. Cumulative lifetime steroid exposure was calculated and scored as previously described, utilizing 20 g as a cutoff for high cumulative exposure in an adult or child heavier than 50 kg, or 300 mg/kg in a child below 50 kg [41].

### Statistical analysis

All statistical analyses were performed using R statistical package [47]. cOPN was the primary measure in this study and data that were not normally distributed were log-transformed when required, in order to fit major assumptions of parametric statistical models in analysis. Differences between two groups were examined initially using Student's *t*-test for continuous variables, and Fisher's exact test for categorical variables. Correlations between two groups were evaluated using the Pearson test for parametric variables. Univariate analyses were performed to assess the effect of variables on AMS and SDI; in order to estimate effect size multiple logistic regression was performed and this included variables previously associated with SDI, particularly in pSLE [5,6,48]. *P*-values ≤ 0.05 were considered statistically significant.

## Results

### Demographics of the pSLE and aSLE cohorts

Forty-two individual pSLE patient plasma samples with one year of subsequent complete clinical data were included for analysis. The mean age of diagnosis of the pSLE patients was 12.7 ± 4.1 years, with an age of enrollment of 15.9 ± 3.5 years (demographic and disease characteristics; Table 1). The pSLE cohort included twenty-one patients (50%) who self-identify as Hispanic, ten (24%) as Asian, five (12%) as Caucasian (non-Hispanic), four (9%) as African-American, and two (5%) identify as other/mixed background. The aSLE cohort was differently distributed, with thirteen (56%) of the patients identifying as Caucasian (non-Hispanic) and the remainder of the cohort comprised of two patients (9%) who self-identify as Hispanic, two as Asian, four (17%) as African-American, and two who identify as other/mixed background. Although both the pSLE and aSLE cohorts were established at the same urban tertiary referral center, discordant ethnic/racial representation may reflect differences in insurance status. In California, all financially eligible pSLE patients under age 21 years qualify for state-administered health insurance, whereas most patients with SLE over age 21 years do not qualify for similar benefits.

**Table 1:** Baseline characteristics of patients in the longitudinal pediatric- and adult-onset systemic lupus erythematosus (pSLE and aSLE) cohorts*

	pSLE	Pediatric controls	aSLE	Adult controls
Sex, ratio, male:female,	10:32	10:12	2:21	16:24
Ethnicity/race, n (%)				
Hispanic	21 (50)	5 (23)	2 (9)	6 (15)
Asian	10 (24)	10 (45)	2 (9)	13 (32)
Caucasian	5 (12)	6 (27)	13 (56)	16 (40)
African-American	4 (9)	1 (5)	4 (17)	4 (10)
Other/Mixed	2 (5)	0	2 (9)	1 (3)
Age, years	15.9 ± 3.5	20.2 ± 1.3	42 ± 15	41 ± 10
Age at diagnosis, years	12.7 ± 4.1		32 ± 15	
Disease duration, years^†^	3.1 ± 3		7.9 ± 8.6	
SLEDAI, mean (range)	2.0 (0, 15)		3.0 (0, 12)	
SDI, mean (range)	0.9 (0, 5)		0.9 (0, 2)	
Glomerulonephritis, n (%)^††^	30 (71)		5 (22)	
Class II	0		0	
Class III	0		0	
Class IV	17 (40)		4 (17)	
Class V	6 (14)		0	
Mixed	5 (12)		0	

*Except where indicated otherwise, values are expressed as the mean ± SD and were obtained at the time of study entry. SLEDAI, Systemic Lupus Erythematosus Disease Activity Index; SDI, Systemic Lupus International Collaborating Clinics/American College of Rheumatology Damage Index; Glomerulonephritis classification as per the International Society of Nephrology/Renal Pathology Society. There was no biopsy done on one aSLE patient with clinical criteria of SLE renal disease. ^†^*P *= 0.002 in pSLE compared with aSLE; ^††^*P *= 0.0005 in pSLE compared with aSLE.

A range of disease activity was represented at enrollment, however the two cohorts approximated each other with mean SLEDAI score in pSLE of 2 (range 0 to 15) and in aSLE of 3 (range 0 to 12). Similarly, the mean SDI of 0.9 at baseline was the same for the two cohorts (range 0 to 5 in pSLE, and 0 to 2 in aSLE), although the disease duration at enrollment was less in pSLE (3.1 ± 3 years) compared with aSLE (7.9 ± 8.6 years, *P *= 0.002). There was a significant difference in kidney disease, with 71% of the pSLE cohort having renal involvement diagnosed at, or prior to, the initial study visit, compared with 22% in aSLE (*P *= 0.0005).

### Increased circulating plasma OPN levels are associated with pSLE and aSLE

**Figure 1:**
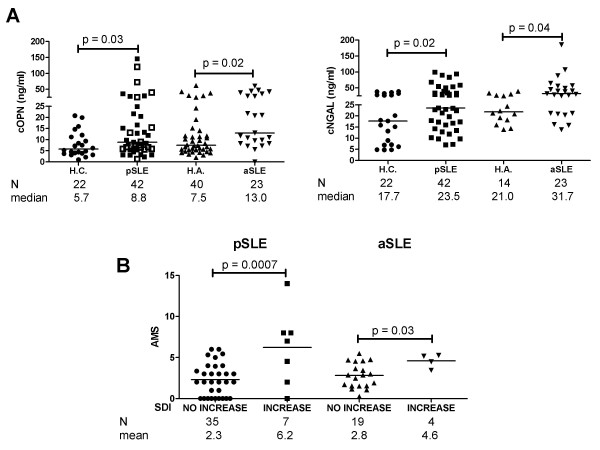
**Indicators of disease and cumulative disease activity in pediatric- and adult-onset systemic lupus erythematosus (pSLE and aSLE)**. **(A) **Circulating plasma osteopontin (cOPN) and neutrophil gelatinase-associated lipocalin (cNGAL) levels in pSLE and aSLE are increased compared with unrelated age-matched healthy young controls (H.C.) and unrelated healthy adults (H.A.), respectively. pSLE patients with active disease are represented by open squares. **(B) **Increased 6-month adjusted-mean SLE disease activity index (AMS) is seen in pSLE and aSLE patients who have an increase in Systemic Lupus International Collaborating Clinics/American College of Rheumatology (SLICC/ACR) damage index scores (SDI) at the end of the 12-month follow up period.

Patients with pSLE were evaluated at baseline for several parameters by cross-sectional analysis. cOPN was increased in the total pSLE sample compared with healthy, unrelated age-matched controls (ages 17 to 22 years; mean ± SD: 20.3 ± 1.3 years): median 5.7 ng/ml in controls vs. 8.8 ng/ml in pSLE (*P *= 0.03) (Figure 1A). Similar results were seen when comparing cOPN in aSLE with healthy, unrelated age-matched controls: median 7.5 ng/ml in controls vs. 13.0 ng/ml in aSLE (*P *= 0.02) Figure 1A). cNGAL was also increased at baseline in both the pSLE cohort and the aSLE cohort compared with the age-matched controls: 17.7 ng/ml in controls vs. 23.5 ng/ml in pSLE (*P *= 0.02); 21.0 ng/ml in controls vs. 31.7 ng/ml in aSLE (*P *= 0.04).

Interestingly, cOPN was slightly increased in the healthy adult controls (ages 25 to 67 years; mean 41 ± 10.6 years) compared with the young healthy controls, although not significantly (median 7.5 ng/ml in adults vs. 5.7 ng/ml in young controls, *P *= 0.18). Although cOPN was higher in the aSLE vs. the pSLE patients (median 13.0 ng/ml vs. 8.8 ng/ml, *P *= 0.04), there was no correlation of cOPN with increasing age in the combined controls (*r *= 0.21, *P*-value = 0.2), or in the combined SLE patients (*r *= 0.07, *P*-value = 0.4). Despite differences in ethnic/racial distribution, no difference was seen when comparing cOPN in Caucasian (non-Hispanic) patients vs. a combined group of Hispanic patients plus patients of African-American, Asian and mixed race descent (cOPN: 19.9 ± 4.5 and 20.3 ± 4.2 ng/ml, respectively, *P *= 0.4)

High IFN-α activity was observed more frequently in pSLE with baseline cOPN in the top vs. the bottom quartile (43% vs. 27%, *P *= 0.02). As in previous studies, pSLE patients with active (SLEDAI ≥ 4) compared with less active disease were more likely to have high IFN-α activity (high IFN-α activity was observed in 50% vs. 18% of pSLE patients with active vs. less active disease, *P *< 0.0001) [49]. However, no association between increased IFN-α activity and SDI was observed (high IFN-α activity was seen 21% vs. 26% of pSLE with SDI scores of 0 vs. > 0, *P *= 0.5).

### Increased cumulative disease activity is associated with increased SDI scores

Bivariate analysis revealed that pSLE and aSLE patients with an increase in SDI score over the course of the study period also had increased AMS, a time-adjusted measurement of cumulative disease activity (pSLE: AMS 2.3 vs. 6.2, *P *= 0.0007; aSLE: AMS 2.8 vs. 4.6, *P *= 0.03) (Figure 1B), confirming previously described results [50]. Overall, there was no difference in AMS in pSLE compared with aSLE (mean AMS 3.4 vs. 3.1, respectively).

### Increased plasma NGAL levels are associated with worsening SLEDAI scores

**Figure 2:**
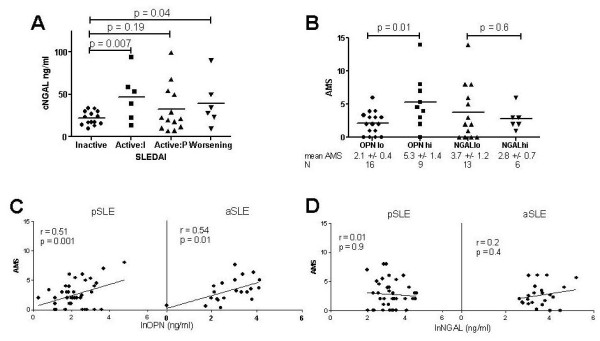
**Association of circulating plasma osteopontin (cOPN), but not circulating neutrophil gelatinase-associated lipocalin (cNGAL) with adjusted-mean systemic lupus erythematosus disease activity index (AMS) at the 6-month interval**. **(A) **Baseline cNGAL in pediatric-onset systemic lupus erythematosus (pSLE) was lower in patients with persistently inactive disease over a 6-month period, but did not differentiate among patients with active disease at baseline, which improves (Active:I); remains persistently active (Active:P); or worsens over 6 months. **(B) **Increased 6-month AMS in pSLE is associated with baseline high cOPN (OPNhi; osteopontin levels in the top quartile) compared with cOPN in the bottom quartile (OPNlo), but not with baseline high cNGAL levels (NGALhi and NGALlo, also defined as top and bottom quartile, respectively). **(C) **cOPN at baseline correlates with 6-month AMS in pSLE (*n *= 42) and in adult-onset SLE (aSLE) (*n *= 23). **(D) **There is no correlation of baseline cNGAL levels in the total pSLE cohort or in aSLE. SLEDAI, SLE disease activity index.

When baseline SLEDAI was evaluated independently of AMS, there was no correlation at the time of blood draw with cOPN (*r *= 0.09, *P *= 0.6) or with cNGAL (*r *= -0.18, *P *= 0.3) in the pSLE sample. Conversely, there was an association of increased cNGAL with worsening SLEDAI in pSLE (Figure 2A); the threshold between persistently inactive or active disease was characterized by a SLEDAI of 4 over the 6-month period, and improved or worsening was defined as a threshold SLEDAI change of greater than or equal to 4 compared to baseline. Although there was no association of cOPN with future flare (*P *≥ 0.6) in either the pSLE or aSLE longitudinal cohorts, when pSLE and aSLE patients were grouped by highest or lowest quartile cOPN at baseline (See Table 2 for clinical features), mean AMS at 6 months was increased in subjects with high baseline cOPN (pSLE: 5.3 vs. 2.1, *P *= 0.01; aSLE: 4.6 vs. 2.7, *P *= 0.01) (Figure 2B), but not in subjects with baseline cNGAL in the top quartile (Figure 2B).

**Table 2:** Baseline clinical features of pediatric- and adult-onset systemic lupus erythematosus (pSLE and aSLE) patients based on osteopontin (OPN) quartiles*

	pSLE			aSLE		
	
	OPNlo	OPNhi	*P*-value	OPNlo	OPNhi	*P*-value
Current prednisone dosage, mg/day, median (range)	10 (0, 60)	30 (0, 40)	0.6	5 (0, 5)	12.5 (0, 60)	0.1
High cumulative prednisone dose, patients, number (%)	2 (13)	8 (73)	0.01	1 (25)	3 (27)	0.3
Current immunosuppressive use, patients, number (%)	7 (44)	7 (64)	0.6	2 (25)	5 (46)	0.6
Renal involvement, patients, number (%)	8 (50)	8 (73)	0.4	0 (0)	4 (36)	0.5
Disease duration, years	2.3 ± 2.7	4.5 ± 3.5	0.2	8.5 ± 12.3	11.8 ± 7.6	0.7
SDI, median (range)	0 (0, 3)	2 (0, 5)	0.01	0 (0, 2)	0 (0, 2)	0.9
SLEDAI, median (range)	2 (0, 8)	2 (0,16)	0.3	2 (0, 8)	4 (0, 8)	0.6
ESR	18 ± 7	54 ± 28	0.01	8 ± 6	45 ± 35	0.1
C3	100 ± 32	95 ± 40	0.7	115 ± 15	89 ± 24	0.9
C4	15 ± 5		0.7	22 ± 3.3	19.2 ± 5.5	0.3
αdsDNA, % patients negative/% patients positive	46/8	40/40	< 0.0001	100/0	43/29	< 0.0001
αRBP, % patients positive	25	45	0.003	20	43	0.0005

*Except where otherwise indicated, values are expressed as mean ± standard deviation. The lowest and highest quartile of OPN (OPNlo and OPNhi) were defined as circulating plasma OPN levels < 7.8 ng/ml (*n *= 16 in pSLE and 11 in aSLE) or > 19.1 ng/ml (*n *= 4 in pSLE and 11 in aSLE), respectively. High cumulative prednisone dose was defined as > 300 mg/kg in pSLE and > 20 g in aSLE. SDI, Systemic Lupus International Collaborating Clinics/American College of Rheumatology (SLICC/ACR) damage index; SLEDAI, SLE, disease activity index; ESR, erythrocyte sedimentation rate; C3 and C4, complement 3 and 4; αdsDNA, anti-dsDNA antibody; borderline positive results were not included. αRBP, anti-RNA binding protein antibody.

### High plasma OPN levels are associated with autoantibody profile

Patients with pSLE and aSLE were grouped by bottom and top quartile of cOPN for comparison at baseline. Patients who were positive for antibodies to dsDNA at study enrollment were more likely to have high cOPN (*P *< 0.0001 for pSLE and aSLE) (Table 2). Similarly, the percentage of patients that demonstrated positivity for antibodies to RNA-binding proteins (anti-SSA/SSB and/or anti-Sm/RNP antibodies) was higher in the group of patients with high cOPN (*P *= 0.003 and 0.0005 in pSLE and aSLE, respectively) (Table 2). The mean ESR was higher in patients with high cOPN (*P *= 0.01 and 0.1 in pSLE and aSLE, respectively) (Table 2), despite no difference in SLEDAI or complement levels among these groups. Overall, there was a higher amount of cumulative (lifetime) prednisone exposure among the pSLE patients with high cOPN (*P *= 0.01) (Table 2). Furthermore, although the portion of patients with renal involvement was higher in the high cOPN quartile vs. low cOPN, these differences did not reach statistical significance (8/11 and 8/16, respectively in pSLE; 4/11 and 0/4, respectively in aSLE) (Table 2).

### Increased circulating plasma OPN levels precede increased 6-month cumulative disease activity and 12-month SDI changes in pSLE and aSLE

Increased cOPN at baseline correlated with increased AMS over the subsequent 6 months in pSLE (*r *= 0.51, *P *= 0.001) (Figure 2C), as well as in aSLE (*r *= 0.52, *P *= 0.01). A subgroup analysis of pSLE patients with renal involvement (*n *= 32) demonstrated similar results (*r *= 0.44, *P *= 0.02); cOPN in renal patients with active renal SLEDAI scores (*n *= 13; scores for renal categories of the SLEDAI assessment ≥ 4) showed a trend towards increased levels (26 ng/ml vs. 12 ng/ml, *P *= 0.1), suggesting increased cOPN is present in patients with renal flare. Correlation between cNGAL and AMS was not observed (Figure 2D).

**Figure 3:**
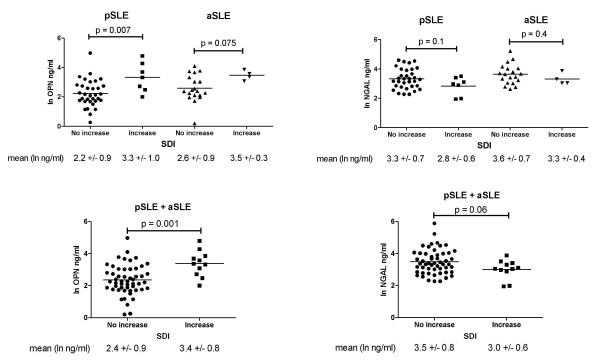
**Baseline circulating osteopontin levels correlated with disease-related damage accumulated over the subsequent twelve months**. Mean baseline natural log-transformed circulating osteopontin (lncOPN) was higher in pediatric- and adult-onset systemic lupus erythematosus (pSLE and aSLE) patients who had an increase in (SDI) scores over the 12-month study period (left), but not mean natural log-transformed circulating neutrophil gelatinase-associated lipocalin (lncNGAL) (right). SDI, Systemic Lupus International Collaborating Clinics/American College of Rheumatology (SLICC/ACR) damage index.

At the baseline visit, bivariate analysis revealed pSLE patients with any organ damage (SDI > 0; *n *= 16) had increased cOPN levels compared with pSLE patients with no damage (median 18.5 ng/ml in SDI > 0 vs. 6.6 ng/ml in SDI = 0 at enrollment, *P *= 0.02). In addition, SDI scores at enrollment correlated with increased cOPN (*r *= 0.43, *P *= 0.005). Of 42 pSLE patients, 7 (16%) had an increase in SDI over the 12-month study period, which was also associated with increased baseline cOPN (*P *= 0.007) (Figure 3, top left panel); a similar trend was seen in aSLE patients (*P *= 0.075) (Figure 3, top left panel) and a combined analysis of pSLE with aSLE demonstrated statistical significance (*P *= 0.001) (Figure 3, bottom left panel). There was no difference in baseline cNGAL in pSLE or aSLE with an increased SDI, although a combined analysis of pSLE and aSLE demonstrated a trend of lower cNGAL at baseline in those individuals with an increase in SDI over the study period (Figure 3, right panels).

### Increased circulating plasma OPN may predict cumulative disease activity and risk for increase in SDI in pSLE

Univariate logistic regression analysis (Table 3) did not identify any potential factors in this cohort as associated with high cumulative SLE disease activity (AMS > 4). However, on univariate and multivariate analysis, high cOPN was associated with an increase in SDI at 12 months (odds ratio (OR) 7.5, 95% CI 2.9, 20, *P *= 0.03). Utilizing a high cOPN cutoff of the bottom three quartiles of healthy controls of all ages (*n *= 62) or 2 SD above the mean for young, healthy controls (*n *= 22) yielded identical results; the groups of normal versus high cOPN in this study are not altered by the change in the cutoff. The area under the curve (AUC), when generating the receiver-operating characteristic (ROC) of baseline cOPN sensitivity and specificity for the indication of SLE patients with an increase of SDI over a 12-month period, is 0.543 (95% CI 0.347, 0.738, positive predictive value 95%, negative predictive value 38%).

**Table 3:** Univariate and multivariate logistic regression analysis of potential risk factors during 12-month follow up in pediatric-onset systemic lupus erythematosus (pSLE)*

			Univariate analysis		Multivariate analysis	
			
Variable	AMS < 3.7	AMS > 3.7	OR (95% CI)	*P*-value	OR (95% CI)	*P*-value
Osteopontin high	8	3	2.1 (0.39, 10.8)	0.4		
NGAL high	7	2	0.8 (0.14, 4.8)	0.9		
Cumulative prednisone exposure	12	3	0.7 (0.15, 3.2)	0.7		
Disease duration > 2 years	16	4	0.6 (0.15, 3.2)	0.7		
Renal involvement	22	7	1.4 (0.3, 8.3)	0.9		
Non-Caucasian background	25	10	5.4 (0.28, 104)	0.3		
Male gender	8	2	0.8 (0.04, 3.1)	0.9		
						
	ΔSDI = 0	ΔSDI > 0				
Osteopontin high	6	4	9 (1.5, 60)	0.015	7.5 (2.9, 20)	0.03
NGAL low	8	2	1.3 (0.21, 8)	0.9	1.16 (0.14, 9.3)	0.9
Cumulative prednisone exposure	11	4	2.8 (0.5, 14.7)	0.4		
Disease duration > 2 years	17	2	0.4 (0.08, 2.4)	0.4		
Renal involvement	24	5	2.1 (0.2, 20.2)	0.9		
Non-Caucasian background	28	7	3.4 (0.2, 68)	0.6		
Male gender	10	0	0.6 (0.008, 3)	0.16		

*Except where indicated otherwise, values reflect the number (%) of patients. Odd ratios (ORs) were determined for each variable based on a yes/no determination in patients with pSLE (*n *= 42). Potential risk factors identified by univariate analysis (*P *≤ 0.1) were included in the multivariate models. Total number of SLE patients with adjusted mean SLE disease activity index (AMS) in the top quartile (AMS > 3.7) is 10; total number of SLE patients with change in SDI (ΔSDI) greater than 0 is 7. SDI, Systemic Lupus International Collaborating Clinics/American College of Rheumatology (SLICC/ACR) damage index; NGAL, neutrophil gelatinase-associated lipocalin.

## Discussion

In this study, we describe the potential for circulating plasma OPN levels to determine the odds of accruing organ damage in SLE after one year. We have shown that increased cOPN precedes increased cumulative disease activity in SLE and an increase in disease damage score, especially in pSLE. This relationship with cumulative disease activity and increased damage score was not seen with cNGAL, however, increased baseline cNGAL was associated with a worsening SLEDAI at the subsequent clinic visit, as expected based on previous reports [8]. Following an analysis of possible factors associated with an increase in SDI over 12 months, cOPN was identified as potentially useful in identifying pSLE patients at risk for serious outcomes. Although a trend of lower baseline cNGAL levels was seen in SLE patients with an increase of SDI at one year, low cNGAL levels were not associated with increased cumulative disease activity or SDI increase on univariate or multivariate regression analysis. The findings from this study suggest the use of cOPN as a marker of poor outcome in pSLE during 12-month follow up, and may have broader implications for its use in assessing the odds of poor prognosis of SLE at any age. The value of cOPN is unique as a possible biomarker for risk of disease damage in SLE; in this role it is providing additional information by identifying patients who would require closer clinical follow up over time, in comparison with established biomarkers of impending disease flare such as NGAL.

The prediction of risk for irreversible damage could be critical for guiding treatment decisions in patients with SLE, and particularly in pSLE patients who have increased rates of organ damage and resultant higher cost of care compared with adult patients [4-6,51-53]. In clinical practice, the testing of cOPN may have the potential to be more practical to clinicians for predicting damage compared to AMS, a labor-intensive calculation. We used AMS as the scale for cumulative disease activity due to its established value in the prediction of organ damage, our primary outcome measure, in both adult and pediatric-onset SLE [6]. We confirmed in our study that increased AMS is associated with increases in SDI, and importantly, uniquely report that increased cOPN is similarly associated with increases in SDI. An assay such as peripheral OPN, which may quickly and easily determine that a patient with SLE is at higher risk for worse outcome, could lead to more effective categorization of patients in clinical practice.

The limitation of this study is the modest size of the cohort and the duration of follow up. Longer follow up periods in larger groups will help determine the true predictive ability of cOPN in SLE outcomes over time. Although it was not the primary intention of this study to determine whether cOPN fluctuates with disease activity or flare, future study should include a score that captures changes in disease activity over time, such as the British Isles Lupus Assessment Group index, or include novel pSLE flare criteria; particular attention should be given to the renal domain as a strong correlation was seen between increased AMS and cOPN in a subgroup of our SLE patients with renal involvement [54]. We did not see differences in mean cOPN between Caucasian and non-Caucasian SLE patients. Larger longitudinal studies would permit the stratification of patients by race/ethnicity, as well as by potentially important modifiers such as specific organ involvement, presence or absence of organ damage, and disease duration at baseline.

Interestingly, a previous comparison by Marhaug *et al. *showed higher cOPN in healthy children compared with adults [55]. In our healthy adult sample, there appeared to be a right skew in the distribution of cOPN. There was no overall correlation in our adult sample between cOPN and increasing age however, suggesting that age alone is not a factor for increased cOPN. In fact, using a cutoff for high cOPN based on 2 SD above the mean for young, healthy controls (whose cOPN levels were distributed normally) did not change the results, even when including the adult controls in the analysis. It is possible that potential confounders for increased cOPN exist in the age range of our healthy adults for whom full medical records were not available, such as subclinical coronary artery disease [56]. Although patients with SLE are prone to premature atherosclerosis, it is likely that younger patients with SLE may have less atherosclerosis due to their young age and usually relatively shorter disease duration; furthermore, cOPN may be an indicator of global disease burden and not driven solely by cardiovascular inflammation. As we expand the cohort and continue to study the ability of cOPN in determining risk for SLE disease progression, we may be able to identify those confounders that increase cOPN, including cardiovascular disease, medication use, and baseline individual characteristics, such as ancestry or gender, and thus improve the positive predictive value of the cOPN test, which was calculated at 95% [57]. The observed 95% positive predictive value of a high cOPN test for damage due to SLE in the subsequent 12 months warrant a follow up study using a larger, independent longitudinal SLE cohort.

In our study, we have confirmed increased cOPN in pSLE compared with healthy, unrelated controls, and an enrichment of anti-dsDNA and anti-RBP antibodies in SLE patients with high cOPN. A genetic basis for familial correlation of cOPN has not been confirmed in large-scale genome-wide association studies of SLE genetic susceptibility. However, *OPN *polymorphisms predispose to increased OPN expression in Europeans and high serum IFN-α activity in younger SLE patients, even though to our knowledge, *OPN *has not been reported as a IFN-α-regulated gene per se [14,58]. OPN may play a role in SLE progression once disease is established, even if it may not be a risk factor for initial development of autoimmunity. The interaction of intracellular OPN with the TLR7/9 pathway leading to the production of IFN-α in murine plasmacytoid dendritic cells and the presence of anti-dsDNA antibodies in OPN-expressing transgenic mice suggests the importance of this molecule to autoimmunity [16,59]. Suppression of murine Th17 by IFN-α receptor signaling was shown to be mediated by inhibition of OPN expression [17]. OPN can be induced by several inflammatory cytokines, including IL-1β, IFN-γ and TNF-α [60]. Extracellular OPN enhances macrophage migration, survival and cytokine production, playing a critical role in chronic inflammation [23,35,61,62]. Alternatively activated macrophages express OPN and may be key regulators of crescentic lupus nephritis, asthma, and scleroderma lung disease [30,37,39]. Although cOPN may not be a specific marker for SLE, it may be a more upstream event compared with biomarkers of flare such as NGAL; therefore, it may have value as a marker for cumulative disease progression resulting in organ damage.

## Conclusions

To our knowledge, this is the first study to show that circulating plasma OPN may have potential as a predictor of increased cumulative disease activity and risk of organ damage in pSLE. Our data raise the possibility that the presence of OPN heralds the onset of a process that may ultimately lead to irreversible tissue damage. OPN functionally contributes to inflammation-associated fibrosis via macrophage recruitment in acute and chronic inflammation, potentially participating in an environment of dysregulated tissue repair in SLE-affected organs [23,24,63-65]. Further studies are needed to validate our results in the context of both longitudinal observational cohorts and randomized clinical trials, and to confirm the specific roles of OPN in the process of organ damage in SLE.

## Abbreviations

ACR: American College of Rheumatology; AGP: alpha1-acid-glycoprotein; AMS: adjusted mean systemic lupus erythematosus disease activity index; anti-dsDNA: anti-double stranded DNA; aSLE: adult-onset systemic lupus erythematosus; AUC: area under the curve; C3: complement; cNGAL: circulating plasma Neutrophil gelatinase-associated lipocalin; cOPN: circulating plasma osteopontin; ELISA: enzyme-linked immunosorbent assay; ESR: erythrocyte sedimentation rate; IL: interleukin; ISN/RPS: International Society of Nephrology/Renal Pathology Society; L-PGDS: lipocalin-type prostaglandin-D synthetase; MCP-1: monocyte chemotactic protein-1; M-CSF: macrophage colony stimulating factor; NGAL: neutrophil gelatinase-associated lipocalin; OPN: osteopontin; OR: odds ratio; pSLE: pediatric-onset systemic lupus erythematosus; RANTES: regulated on activation, normal T expressed and secreted; ROC: receiver-operating characteristic; SDI: Systemic Lupus International Collaborating Clinics/American College of Rheumatology (SLICC/ACR) damage index; SELENA: Safety of Estrogens in Lupus Erythematosus: National Assessment; anti-Sm/RNP: anti-Smith/Ribonucleoprotein; SLEDAI: systemic lupus erythematosus disease activity index; TNF: tumor necrosis factor; TRAP: tartrate-resistant acid phosphatase; UCLA: University of California, Los Angeles.

## Competing interests

The authors have no competing interests.

## Authors' contributions

OR and JW contributed to study conception and design, acquisition of data via immunoassays and clinical data collection, performed data analysis and interpretation, and drafted the manuscript. MP, AH, TN, JG and BH contributed to acquisition and analysis of the clinical data, including scoring of disease activity. PM and DE provided statistical analysis and interpretation for the study. MM and DK contributed to study conception and design and data interpretation. BT contributed to study conception and design, assisted with data interpretation, and helped draft the manuscript. All authors have read, revised, and approved the final manuscript.
